# Interventions to increase adherence to micronutrient supplementation during pregnancy: a protocol for a systematic review

**DOI:** 10.1111/nyas.14319

**Published:** 2020-02-13

**Authors:** Filomena Gomes, Gilles Bergeron, Megan W. Bourassa, Diana Dallmann, Jenna Golan, Kristen M. Hurley, Shannon E. King, Ana Carolina Feldenheimer da Silva, Saurabh Mehta

**Affiliations:** ^1^ The New York Academy of Sciences New York City New York; ^2^ School of Human Nutrition McGill University Montreal Quebec Canada; ^3^ Division of Nutritional Sciences Cornell University Ithaca New York; ^4^ Johns Hopkins Bloomberg School of Public Health Baltimore Maryland; ^5^ Vitamin Angels Santa Barbara California; ^6^ Department of Nutrition in Public Health the University of State of Rio de Janeiro Rio de Janeiro Brazil

**Keywords:** pregnancy, micronutrients, supplementation, adherence, compliance

## Abstract

Micronutrient supplementation during pregnancy has been shown to be a cost‐effective method to reduce the risk of adverse pregnancy and birth outcomes. However, one of the main barriers to the successful implementation of a micronutrient supplementation program in pregnancy is poor adherence. Our review will assess the effectiveness of interventions designed to increase adherence to micronutrient supplements in pregnancy. Following the Cochrane Collaboration Methodology, we will start by conducting the literature searches on Medline (via PubMed), Embase, Scopus, Web of Science, and Cochrane Library, in addition to sources of gray literature, to retrieve all the available relevant studies. We will include randomized controlled trials and nonrandomized studies with a control group, where participants are pregnant women taking any micronutrient supplements in the context of antenatal care globally. We will include studies with targeted interventions designed to improve adherence to micronutrient supplementation in pregnant women compared with (1) usual care or no intervention or (2) other targeted micronutrient adherence intervention. Abstract selection, data extraction, and risk of bias assessment (according to the type of studies) will be conducted by two independent reviewers. The pooled results will be reported using the standardized mean differences for continuous data, and odds ratio or risk ratio for dichotomous data. We will assess sources of heterogeneity and publication bias. By following this protocol, we will systematically assess and synthesize the existing evidence about interventions designed to increase adherence to micronutrient supplementation in pregnant women. Understanding which strategies are more effective to increase the consumption of micronutrient supplements during this critical stage of life will have significant implications for clinicians and policymakers involved in the delivery of prenatal micronutrient supplementation interventions.

## Introduction

Adequate nutrition is a cornerstone of good health through the life cycle, but is particularly important during pregnancy, for maternal health, and fetal development. Many micronutrients, especially vitamins A, D, E, B_6_, B_9_ (folate/folic acid), B_12_, and C, and the minerals iron, zinc, iodine, copper, and selenium, are critical during pregnancy and the recommended intakes for most of these nutrients increase by as much as 50% during pregnancy to accommodate higher maternal, placental, and fetal requirements.^1^, ^2^ Unfortunately, micronutrient deficiencies in pregnancy remain widespread globally, particularly in low‐ and middle‐income countries (LMIC), as a result of women entering pregnancy malnourished, combined with high rates of infections and the increased nutritional demands of pregnancy. Consequently, micronutrient deficiencies in pregnancy are associated with adverse pregnancy and birth outcomes, such as maternal mortality, pregnancy loss, birth defects, low birth weight (LBW), risk of death in infancy, stunting, and may influence long‐term outcomes for the offspring, such as cognitive development and cardiometabolic risk.^1^, ^3^, ^4^ Nevertheless, prenatal micronutrient supplementation can efficaciously address these micronutrient gaps and, in turn, reduce the risk of negative pregnancy and birth outcomes. Prenatal multiple micronutrient supplements, containing 15 vitamins and minerals, have been shown to be a cost‐effective method to decrease the risk of stillbirth, LBW, preterm birth (PB), and being born small‐for‐gestational‐age.^3^, ^5^ There are a number of examples of efficacious individual micronutrient interventions, including folic acid to prevent neural tube defects, iodine to prevent congenital hypothyroidism, zinc to reduce the risk of PB, calcium to reduce the risk of preeclampsia, and iron to reduce the risk of iron‐deficiency anemia and LBW.^1^, ^2^

Despite the availability of efficacious interventions, poor adherence remains a main barrier to the successful implementation of micronutrient supplementation programs. Even when a program has high coverage (i.e., the women are receiving the supplements), low adherence prevents pregnant women and their unborn babies from receiving the maximum potential benefits of this intervention.^6^ Data from Demographic and Health Surveys from 22 LMIC showed that while coverage of antenatal care visits and provision of iron and folic acid tablets was higher than 80% (83% and 81%, respectively), only 8% of women who purchased or received the supplements consumed the recommended dose, which the study authors defined as being 180 tablets or more of these supplements.^6^ Other study authors who used a lower and more common cutoff to define reported adherence to prenatal micronutrient supplements (i.e., 90 tablets) found similar values of poor adherence, varying between 0.6% and 11.7%, depending on the type of micronutrient supplement analyzed.^7^

In health care, adherence has been defined as “the extent to which a patient's behavior matches the agreed recommendations from a healthcare provider.”^8^ While awareness and knowledge are known to influence the adoption of positive health behavior,^9^ other important factors may play an important role in the behavior change of the pregnant mother (e.g., forgetfulness, lack of time, age group, level of education, unplanned pregnancy, high cost, side effects, or simply the difficulty in taking tablets).^10^, ^11^, ^12^ Unfortunately, health behavior is difficult to change and low adherence prevents pregnant women and their fetuses from receiving the potential maximum benefits of supplementation. Thus, there is a need to identify strategies that help increase adherence to micronutrient supplements during pregnancy. In fact, this topic was recognized as a top priority in a research prioritization exercise recently conducted by a group of international experts in nutrition and maternal health.^13^ Examples of these strategies include individual counseling, training healthcare professionals, reminders through text messages, financial incentives, family and peer support, among others.

Other authors attempted to address this research gap by conducting a systematic review of interventions to increase awareness, knowledge, and consumption of folic acid before and during pregnancy.^9^ However, this study was conducted more than 10 years ago, limited the inclusion of studies published between 1992 and 2005, was focused on the use of a single micronutrient, and most interventions were delivered at the population level (e.g., mass media campaigns). By contrast, we aim to systematically assess and synthesize all existing evidence about targeted interventions designed to increase adherence to any micronutrient supplementation (in the context of antenatal care) during pregnancy.

## Objectives

The objective of our review is to assess the effectiveness of interventions designed to increase the adherence to micronutrient supplementation during pregnancy.

## Methods

This systematic review will follow the Cochrane Collaboration Methodology^14^ and the Preferred Reporting Items for Systematic Reviews and Meta‐Analyses (PRISMA) reporting guidelines.^15^ A PRISMA flow diagram will be used to illustrate the results of the literature searches and the study screening and selection process. The protocol has been registered with the International Prospective Register of Systematic Review (PROSPERO), the University of York Centre for Reviews and Dissemination (https://www.crd.york.ac.uk/prospero/; registration number CRD42019146814).

### Criteria for considering studies for this review

#### Types of studies

We will include randomized controlled trials (RCTs) and nonrandomized studies with a control group. Studies without a comparison group will be excluded from our review.

#### Types of participants

This review will include studies where participants are pregnant women taking any micronutrient supplement in the context of antenatal care, in a variety of settings (low‐, middle‐, and high‐income countries (LIC, MIC, and HIC, respectively)). Pregnant women who are institutionalized will be excluded.

#### Types of interventions

This review will include studies that use targeted interventions designed to improve adherence to micronutrient supplements in pregnant women, focusing on the intake of the recommended regimen as the outcome. Examples of these interventions include individual counseling, training of healthcare professionals, reminders through mobile phone text messages, financial incentives, family and peer support, among others. It will include any micronutrient or combination of micronutrients provided as a powder, liquid (e.g., syrups and suspensions), or a pill/tablet, for any duration and frequency. One targeted adherence intervention (to micronutrient supplements) will be compared with (1) usual care or no intervention (defined as no intervention aimed at improving adherence to micronutrient supplements), or (2) other targeted micronutrient adherence intervention. In order to determine the isolated effect of the adherence intervention (on consumption of the recommended supplement by the pregnant women), the supplementation regimen provided in different groups needs to contain the same type and dose of micronutrient, and have the same duration (e.g., 30 mg of iron/day for 6 months). The review will exclude interventions delivered at the population level (e.g., mass media campaigns) as they lack an appropriate control group, and interventions that deliver micronutrients through fortified or enriched foods (including micronutrient powders for the point‐of‐use fortification of foods and lipid‐based nutrient supplements) as the behaviors required for adherence differ greatly compared with supplementation.

#### Types of outcome measures

The primary outcomes of interest are adherence to micronutrient supplements (i.e., consumption of the recommended supplementation regimen, as defined by the study authors) and adverse gastrointestinal symptoms (nausea, vomiting, and diarrhea). Additional outcomes of interest will include other adverse effects and pregnancy and birth outcomes (e.g., preeclampsia, anemia, LBW, PB, etc.).

Different systematic reviews use varying cutoffs to define adherence to micronutrient supplements during pregnancy, ranging from 70%^16^ to 95%^17^ of the recommended daily supplementation dose, and the use of a specific threshold to define medication adherence has been criticized.^18^ For these reasons, in our systematic review, we will report adherence as measured and defined by the study author. This crucial piece of information will be provided in the table of characteristics of included studies.

### Search methods for identification of studies

The literature searches will be conducted in five electronic bibliographic databases (Medline (via PubMed), Embase, Scopus, Web of Science, and Cochrane Library), from inception to the date of the searches, with no restrictions on language or date of publication, to retrieve all the available studies. The search terms (using a combination of MeSH terms, free‐text words, and Boolean operators) and search strategies for each database have been defined with assistance from the Cornell University librarians. Additionally, we will also search trial registers (e.g., ClinicalTrials.gov) to identify ongoing studies and the gray literature will be searched using the WHO Library and SciELO.

The bibliographic software Zotero (version 5.0.75) will be used to store, organize, and manage all the references. Study deduplication efforts will also initially occur in Zotero prior to further deduplication in the Covidence systematic review software.

### Data collection and analysis

#### Selection of studies

Titles and abstracts of studies retrieved using the predefined search strategies and those from additional sources (e.g., gray literature and trial registries) will be screened independently by two review authors to identify studies that meet the inclusion criteria defined above. All the retrieved titles and abstracts will be evaluated using Covidence. Any disagreement between the two review authors will be resolved through discussion with a third independent reviewer. The full text of the potentially eligible studies will be retrieved and independently assessed for eligibility (to determine if these criteria set for inclusion) by two review team members, and reasons for inclusion or exclusion will be documented in Covidence. Finally, data will be extracted from all articles that meet the inclusion criteria.

#### Data extraction and management

A standardized data extraction form will be developed to use on each of the included studies, which will later be used for assessment of study quality and evidence synthesis. Extracted data will include study design, study setting (LIC, MIC, and HIC, as per the World Bank classification), study population and participant demographics, baseline characteristics (e.g., age, gestational age at enrollment, and parity), type and form of micronutrient supplement provided, details of the intervention designed to increase adherence and control group description (including the number of participants in each arm), recruitment and study completion rate, how and when adherence was assessed, how the effect of the intervention was measured (e.g., rates of adherence or change in adherence pre‐ and postintervention), and information for the assessment of risk of bias. Study authors will be contacted when important data missing or when more information and details are needed. The extraction of data will be conducted independently by two review authors. Discrepancies will be identified and resolved through discussion. A third author will participate in the discussions when necessary.

#### Assessment of risk of bias in included studies

We anticipate the inclusion of different types of study designs, that is, RCTs and nonrandomized studies with a controlled group (e.g., a controlled before and after study).

The risk of bias for randomized control trials will be independently assessed by two review authors using the following criteria: random sequence generation; allocation concealment; blinding of participants, personnel, and outcomes; incomplete outcome data; selective outcome reporting; and other sources of bias, in accordance with methods recommended by the Cochrane Collaboration,^14^ using the RevMan software (version 5.3). Disagreement in the assessment of risk of bias between the review authors will be resolved by discussion with a third review author.

The risk of bias for nonrandomized studies with a control group will be assessed with the tool “ROBINS‐I” (Risk of Bias in Non‐Randomized Studies of Interventions).^19^ This tool includes the assessment of seven risk of bias domains: the first two domains cover confounding and selection of participants into the study and address issues before the start of the interventions; the third domain addresses the classification of the interventions themselves; the remaining four domains include biases due to deviations from the intended interventions, missing data, measurement of outcomes, and selection of the reported results.

### Data synthesis and analysis

If we find a sufficient number of similar studies (regarding study design) that meet the inclusion criteria, we will conduct a random‐effects meta‐analysis (or separate meta‐analyses for different types of study design). The results will be presented using forest plots, and the summary results will be reported using the standardized mean differences for continuous data (e.g., percentage of adherence) and odds ratio or risk ratio for dichotomous data (e.g., adherence versus nonadherence). A 95% confidence interval and two‐sided *P*‐values will be calculated for each outcome.

Heterogeneity between the studies in effect measures will be assessed using both the *χ* ^2^ test and the *I* ^2^ statistic. An *I* ^2^ value greater than 50% will be considered as indicative of substantial statistical heterogeneity as per the Cochrane Handbook,^14^ but other forms of heterogeneity (e.g., clinical) will also be considered.

We will also assess whether there is any evidence of publication bias. We will use funnel plots to assess small study effects. Owing to several possible explanations for funnel plot asymmetry, we will interpret results carefully.^20^

If there is an insufficient number of studies with the same study design, instead of performing a meta‐analysis we will provide a narrative synthesis. The narrative synthesis will be structured around the type and range of the effect of intervention (designed to increase adherence), the characteristics of the study population, and the type and form of micronutrient used.

#### Dealing with missing data

We will aim to obtain important missing data from authors, if available, and carefully evaluate important numerical data, such as screened, randomized participants as well as intention‐to‐treat, as‐treated, and per‐protocol populations. We will investigate attrition rates, for example, drop‐outs, losses to follow up, and withdrawals, and critically appraise the issues of missing data and imputation methods.

### Subgroup analysis

If applicable, subgroup analysis will be carried out based on factors that may impact the effect estimates, such as type of setting (e.g., urban versus rural, or HIC versus LMIC); type of micronutrient supplement (e.g., micronutrients required in large amounts, such as calcium versus micronutrients required in small amounts); form/presentation of micronutrient supplement (e.g., tablets versus nontablets); age of the women (e.g., adolescents versus nonadolescents); definition of adherence by the study authors (e.g., self‐reported versus nonself‐reported; less than versus 80% or more of the recommended daily dose); use of behavioral theory to develop the adherence intervention (e.g., yes versus no); duration of the adherence intervention (e.g., <3 versus >3 months); the number of adherence interventions used (e.g., single versus multiple interventions); who delivered the supplements (e.g., medical professional versus community health worker); whether supplements were free or purchased; and whether there is a preexisting health condition (e.g., HIV) or not.

### Sensitivity analysis

Sensitivity analysis may be carried out in order to explore the influence of the following factors (when applicable) on effect sizes:

- Restricting the analysis to very long or large studies to establish the extent to which they dominate the results;
- Restricting the analysis considering risk of bias, as specified in the section “Assessment of risk of bias” in included studies.

## Potential methodological amendments

In the case modifications are required to the present protocol, we will provide a detailed description of what these modifications entailed and a rationale for the need for each change, during the publication of the results of the systematic review.

## Discussion

This protocol was registered and written according to standard guidelines for the conduction of systematic reviews. To our knowledge, this is the first systematic review that will assess the effectiveness of interventions designed to increase adherence to micronutrient supplements in pregnant women. Understanding which strategies can successfully increase the consumption of micronutrient supplements during this critical stage of life will have significant implications for clinicians, policymakers, and program planners involved in the delivery of prenatal supplementation interventions. Ultimately, this will maximize the beneficial impact of such interventions on pregnancy and birth outcomes, as well as financial resources.

## Competing interests

S.M. is an unpaid board member and holds equity in a diagnostic startup focused on the measurement of nutritional biomarkers at the point‐of‐care utilizing the results from his research. All the other authors report no competing interests.
